# Adult-Onset Diffuse Midline Glioma, H3K27-Altered: A Genomics-Guided, Individualized, Multimodal Treatment Approach

**DOI:** 10.3390/brainsci16010097

**Published:** 2026-01-16

**Authors:** Abdussamet Çelebi, Bilal Yıldırım, Emine Yıldırım, Selver Işık, Ezgi Çoban, Erhan Bıyıklı, Osman Köstek, İbrahim Vedat Bayoğlu, Murat Sarı

**Affiliations:** 1 Department of Medical Oncology, Marmara University Faculty of Medicine, Istanbul 34854, Türkiye; abdussametcelebi@gmail.com (A.Ç.); dr-selver83@hotmail.com (S.I.); ezgi.yuzugullu@gmail.com (E.Ç.); osmankostek@yahoo.com (O.K.); dr.vebay@gmail.com (İ.V.B.); 2 Department of Internal Medicine, Marmara University Faculty of Medicine, Istanbul 34854, Türkiye; bilalyldrmctf@gmail.com (B.Y.); cndnyl@gmail.com (E.Y.); 3 Department of Radiology, Marmara University Faculty of Medicine, Istanbul 34854, Türkiye; biyiklierhan@hotmail.com

**Keywords:** diffuse midline glioma (DMG), H3K27-altered, targeted therapy, tissue-agnostic therapy, precision medicine, individualized treatment, dordaviprone (ONC201), trametinib, everolimus, 2-deoxy-D-glucose (2-DDG), electric field therapy, multimodal therapy

## Abstract

**Background**: H3K27-altered diffuse midline glioma (DMG) is a highly aggressive central nervous system malignancy with limited therapeutic options and poor prognosis. Precision medicine strategies that integrate molecular profiling with individualized treatment selection represent a critical avenue for improving outcomes. **Case presentation**: We describe a 31-year-old woman with H3K27-altered DMG who, after standard chemoradiotherapy, was treated with a personalized, mechanism-guided combination regimen based on her tumor’s molecular profile. Next-generation sequencing identified pathogenic alterations in ATRX, H3F3A, and NF1, with a high NF1 mutation allelic fraction indicating RAS/MAPK pathway activation. Immunohistochemistry demonstrated elevated phosphorylated mTOR consistent with PI3K/AKT/mTOR pathway upregulation. The individualized regimen comprised trametinib and everolimus for dual pathway inhibition, the tissue-agnostic agent dordaviprone (ONC201), metabolic modulation with 2-deoxy-D-glucose, and electric field-based therapy. At seven months, MRI showed approximately a 60% volumetric reduction in the enhancing tumor component, accompanied by marked T2-weighted signal regression. Clinically, the patient remained neurologically intact with a Karnofsky Performance Score of 100%. **Conclusions**: This case illustrates the potential clinical value of a genomics-guided, multimodal treatment strategy in H3K27-altered DMG. The systematic integration of comprehensive molecular profiling with mechanistically rational treatment selection may contribute to meaningful radiological and clinical benefit in this otherwise uniformly fatal disease. These observations support further investigation of individualized, pathway-targeted approaches in prospective studies and N-of-1 trial frameworks.

## 1. Introduction

The 2021 WHO Classification of Tumors of the Central Nervous System placed molecular biomarkers at the center of diagnostic and therapeutic decision making. Within this framework, H3K27-altered diffuse midline gliomas (DMGs) are now uniformly classified as CNS WHO grade 4 tumors, underscoring their aggressive biological behavior and dismal clinical course [1]. These neoplasms arise in midline structures such as the thalamus, brainstem, and spinal cord, with an estimated annual adult incidence of approximately 2.3 cases per million [2]. Despite refinements in radiotherapy techniques and systemic therapies, prognosis remains poor, highlighting the unmet need for novel treatment paradigms [3].

Here, we report an adult patient with H3K27-altered DMG who was managed with a comprehensive, individualized, multimodal treatment strategy. The approach integrated next-generation sequencing (NGS)-guided molecularly targeted therapy, electric field-based treatment, and tissue-agnostic anticancer agents. We discuss the rationale for this combination and its implications for precision oncology in DMG.

## 2. Case Presentation

### 2.1. Clinical Presentation and Diagnosis

**Figure 1:**
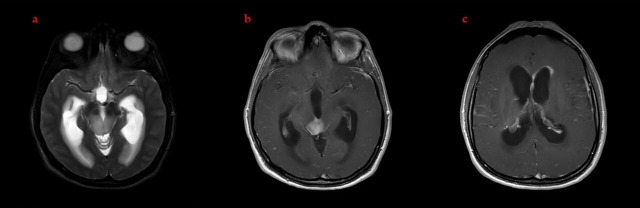
Diagnostic brain MRI findings at presentation. (**a**) Axial T2-weighted image demonstrating a hyperintense mass at the level of the tectal plate–mesencephalon with resultant hydrocephalus secondary to aqueductus cerebri obstruction. (**b**) Axial post-contrast T1-weighted image showing homogeneous enhancement of the lesion. (**c**) Axial post-contrast T1-weighted image demonstrating ependymal enhancement along the frontal horn of the left lateral ventricle and the interventricular septum, consistent with tumor spread.

A 31-year-old woman presented with episodic headaches that were exacerbated by forward flexion. A brain MRI revealed a 22 × 20 mm lesion centered in the tectal plate causing triventricular hydrocephalus. Contrast-enhanced sequences showed heterogeneous enhancement with bilateral thalamic extension, aqueductal obstruction, and small nodular foci along the left frontal horn of the lateral ventricle, raising concern regarding ependymal dissemination. A spinal MRI demonstrated no evidence of leptomeningeal disease (Figure 1).

The patient underwent endoscopic third ventriculostomy (ETV) with tumor biopsy. Histopathological examination revealed a diffusely infiltrative glial neoplasm with mild-to-moderate hypercellularity, moderate nuclear atypia and pleomorphism, and the absence of microvascular proliferation or necrosis. Mitotic activity was elevated at 11–12 mitoses per ten high-power fields, confirmed by phospho-histone H3 (PHH3) immunohistochemistry, with a Ki-67 proliferation index of approximately 15%.

Comprehensive immunohistochemical analysis demonstrated that the neoplastic cells were diffusely positive for glial markers GFAP and OLIG-2, confirming glial lineage. Critically, H3K27M immunohistochemistry showed nuclear positivity in tumor cells with the concurrent focal loss of H3K27me3 trimethylation, a hallmark of H3K27-altered gliomas. (Figure S1) IDH1 immunohistochemistry was negative with appropriate external control, excluding IDH-mutant glioma. The loss of ATRX nuclear expression was observed. Immunohistochemical staining for phosphorylated mTOR (Ser2448) showed increased expression in tumor cells, supporting activation of the PI3K/AKT/mTOR signaling pathway. Markers for neuronal differentiation (synaptophysin, chromogranin, and NeuN) and ependymal differentiation (EMA) were negative.

Next-generation sequencing using a comprehensive CNS tumor panel confirmed the immunohistochemical findings, identifying three Tier-I pathogenic variants: the H3F3A c.83A>T (p.Lys28Met) mutation (variant allele frequency 27.2%, sequencing depth 272×), ATRX c.1443_1452del frameshift deletion (VAF 34.4%, depth 2223×), and the NF1 c.586+1G>C splice site mutation (VAF 64.1%, depth 142×). All the variants were classified as pathogenic according to the ACMG/AMP guidelines, with direct relevance to diffuse glioma pathogenesis. The constellation of H3K27M alteration, ATRX loss, high mitotic activity, and midline location established the diagnosis of H3K27-altered diffuse midline glioma, at WHO grade 4 (File S1).

### 2.2. Definitive Chemoradiotherapy

Given the lesion’s location within eloquent midline structures and the associated surgical risks, the multidisciplinary tumor board concluded that gross total or subtotal resection was not feasible. The patient was treated with definitive radiotherapy (36 Gy in twenty fractions to the entire ventricular system followed by an 18 Gy boost in ten fractions to the enhancing tumor; total dose 54 Gy in thirty fractions) combined with concurrent temozolomide at 75 mg/m^2^ daily. She completed chemoradiotherapy without major acute toxicities.

### 2.3. Post-Treatment Complications

**Figure 2:**
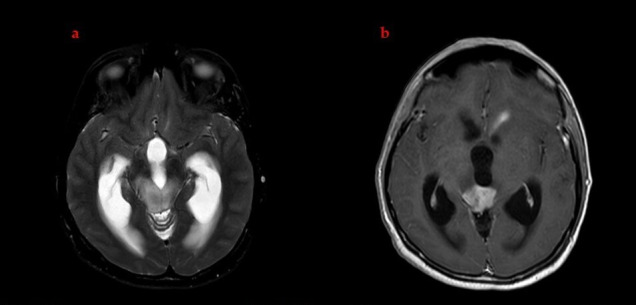
Pre-targeted therapy brain MRI demonstrating the baseline appearance of the lesion. (**a**) Axial T2-weighted image showing the tectal mass accompanied by ventriculomegaly. (**b**) Axial post-contrast T1-weighted image revealing heterogeneous enhancement of the lesion prior to initiation of mechanism-guided targeted therapy.

Several weeks after completing chemoradiotherapy, the patient developed persistent nausea and vomiting. The follow-up MRI showed interval enlargement of the non-enhancing T2-hyperintense tumor component with a worsening hydrocephalus and approximately a 20% increase in the size of the enhancing component, raising concern for ventriculostomy obstruction and possible early progression (Figure 2). A ventriculoperitoneal shunt was inserted. The cerebrospinal fluid cytology was negative for malignant cells. The patient’s symptoms resolved promptly following the procedure, and she was discharged in good clinical condition.

### 2.4. Individualized Multimodal Treatment Strategy

Following the completion of standard chemoradiotherapy and shunt placement, the patient and her family were counseled regarding the limited efficacy of conventional therapies for H3K27-altered DMG. Given the unmethylated MGMT promoter status and concern for early disease progression, the patient was offered both FDA-approved and investigational treatment options. After comprehensive discussion of potential benefits, risks, and the experimental nature of several components, the patient provided informed consent for an individualized, molecularly guided multimodal regimen.

Pathway Analysis and Treatment Rationale: The treatment strategy was developed through the integration of the following: (1) NGS-identified mutations mapped to canonical cancer pathways using OncoKB and CIViC databases; (2) IHC confirmation of pathway activation (phosphorylated mTOR); (3) a literature review of druggable targets in H3K27-altered DMG. The NF1 mutation (VAF 64.1%) was interpreted as likely biallelic inactivation leading to RAS/MAPK activation, providing molecular rationale for MEK inhibition with trametinib.

The regimen comprised the following:

1. Dordaviprone (ONC201): 625 mg orally once weekly (FDA-approved for H3K27M-mutant DMG).
2. 2-Deoxy-D-glucose (2-DDG): 500 mg orally on alternating days as a metabolic inhibitor.
3. Dual pathway inhibition on alternating days:
  - Trametinib 2 mg orally (MEK inhibitor targeting the RAS/MAPK pathway).
  - Everolimus 5 mg orally (mTOR inhibitor targeting the PI3K/AKT/mTOR pathway).

**Figure 3:**
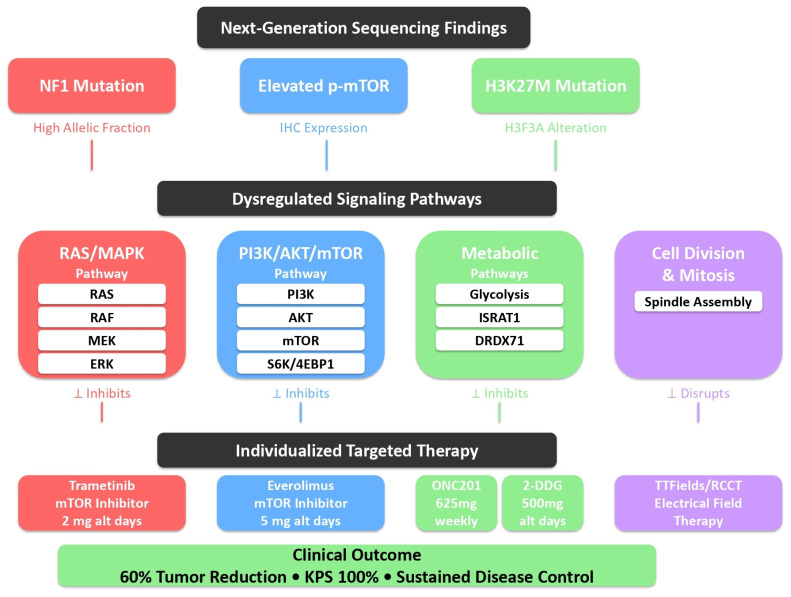
Precision medicine approach: molecular pathways and targeted interventions. Schematic representation of the individualized, mechanism-guided treatment strategy based on comprehensive next-generation sequencing and immunohistochemical profiling. **Top**: Molecular alterations identified through NGS analysis, including NF1 mutation (high allelic fraction), elevated phosphorylated-mTOR expression, and H3K27M mutation (H3F3A alteration). **Middle**: Key signaling pathways activated by these molecular alterations, including the RAS/MAPK pathway (activated by NF1 loss), PI3K/AKT/mTOR pathway (upregulated as evidenced by p-mTOR expression), metabolic pathways (vulnerable due to H3K27M mutation), and cell division/mitosis machinery. **Bottom**: Targeted therapeutic agents selected to address specific molecular drivers and pathway alterations, including trametinib (MEK inhibitor targeting RAS/MAPK pathway), everolimus (mTOR inhibitor targeting PI3K/AKT/mTOR pathway), dordaviprone/ONC201 and 2-deoxy-D-glucose (metabolic inhibitors exploiting tumor metabolic dependencies), and tumor treating fields/ECCT (electric field-based therapy disrupting mitotic spindle assembly). This multi-targeted approach resulted in substantial clinical benefit, including 60% volumetric tumor reduction, sustained disease control, and preserved quality of life (Karnofsky Performance Score: 100%).

This dual-targeted strategy was based on the high allelic fraction of the NF1 mutation, suggesting enhanced RAS/MAPK signaling, and immunohistochemical evidence of increased phosphorylated mTOR, indicating upregulated mTOR activity. The observed PI3K/AKT/mTOR pathway activation was consistent with the known crosstalk between RAS/MAPK and PI3K/AKT/mTOR signaling networks and may have been further potentiated by ATRX loss, which is associated with the alternative lengthening of telomeres and increased mTOR pathway activity. This approach was designed to address convergent oncogenic signaling and prevent compensatory feedback activation between these interconnected pathways (Figure 3).

4. Electro Capacitive Cancer Therapy (ECCT): Non-invasive electric field-based therapy delivered via capacitively coupled electrodes, worn 18–20 h daily according to a standardized protocol.

The overarching treatment strategy was designed to target key molecular drivers identified by NGS while limiting feedback-mediated resistance through concurrent pathway blockades. The regimen incorporated dordaviprone (FDA-approved for H3K27M-mutant DMG) alongside targeted inhibitors selected based on the tumor’s molecular profile. Trametinib and everolimus were used off-label to address the high-fraction NF1 mutation and elevated phosphorylated mTOR expression, respectively. Additional investigational modalities (2-deoxy-D-glucose and electro-capacitive cancer therapy) were included for their CNS penetrance and mechanistic complementarity, administered under close multidisciplinary oversight.

### 2.5. Adverse Events and Management

During treatment, the patient developed a Grade 3 acneiform rash on Day 18 after initiating trametinib (2 mg/day), a recognized class toxicity associated with MEK inhibition (Common Terminology Criteria for Adverse Events [CTCAE] v5.0). The eruption was initially refractory to topical corticosteroids and oral doxycycline over a 2-week period (Days 18–32). The introduction of low-dose systemic isotretinoin (10 mg/day) on Day 33 led to significant improvement within 10 days (by Day 43), allowing the continuation of trametinib without dose reduction. The rash downgraded to Grade 1 by Day 50, and isotretinoin was continued prophylactically throughout the treatment course.

At Week 8 of combination therapy (trametinib + everolimus), the patient experienced Grade 2 thrombocytopenia (platelet count: 68 × 10^9^/L, normal baseline: 245 × 10^9^/L), attributed to everolimus based on temporal correlation and known toxicity profile. Complete blood count (CBC) monitoring was performed weekly during this period. Therapy was interrupted for one week, during which platelet counts recovered to 198 × 10^9^/L (within normal range). Everolimus was then reintroduced at the same dose (5 mg/day) without further hematologic complications; subsequent platelet counts remained stable (range: 180–250 × 10^9^/L) throughout the remaining treatment period, with CBC monitoring every 2 weeks.

No cardiotoxicity, hepatotoxicity, or myelosuppression beyond the aforementioned thrombocytopenia occurred. No unexpected toxicities occurred, and the overall treatment adherence remained high (>95% based on pill counts and electronic device logs).

### 2.6. Treatment Response and Clinical Outcome

**Figure 4:**
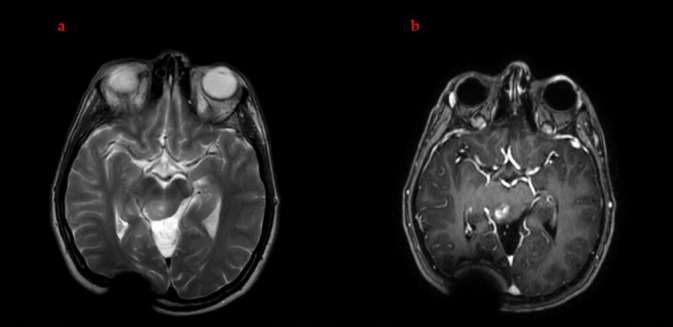
Seventh-month follow-up MRI, demonstrating significant radiological response following initiation of targeted therapy. (**a**) Axial T2-weighted image showing marked regression in the overall tumor dimensions. (**b**) Axial post-contrast T1-weighted image demonstrating nearly a 60% reduction in the enhancing component compared with earlier imaging.

MRI performed seven months after the initiation of the individualized multimodal regimen demonstrated marked radiological improvement. There was substantial regression of the T2-hyperintense tumor component and an approximate 60% volumetric reduction in the enhancing component (Figure 4). These findings were notable in the context of the historical treatment-refractory behavior of H3K27-altered DMG.

**Figure 5:**
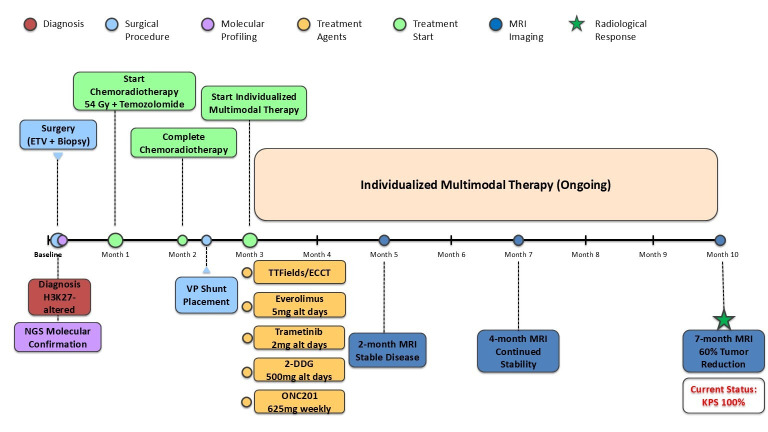
Treatment timeline and clinical course. Timeline illustrating key clinical events, treatment interventions, and imaging assessments from diagnosis through 10 months of individualized multimodal therapy. The patient underwent initial surgery (ETV with biopsy) followed by molecular confirmation via next-generation sequencing. Following completion of standard chemoradiotherapy (54 Gy in 30 fractions with concurrent temozolomide), an individualized multimodal treatment strategy was initiated, incorporating dordaviprone (ONC201), 2-deoxy-D-glucose (2-DDG), dual pathway inhibition with trametinib and everolimus, and electric field-based therapy (TTFields/ECCT). Serial MRI assessments demonstrated progressive tumor response, with 60% volumetric reduction in enhancing tumor at 7 months. The patient remains neurologically intact with a Karnofsky Performance Score of 100%.

Clinically, the patient remained neurologically asymptomatic. At the most recent follow-up, her Karnofsky Performance Score was 100%, reflecting full functional independence and preserved quality of life (Figure 5).

## 3. Discussion

### 3.1. Molecular Pathogenesis and Therapeutic Implications

H3K27-altered DMGs represent a distinct molecular subset of CNS tumors, characterized by recurrent histone H3 mutations [4]. The H3K27M mutation causes global epigenetic dysregulation through the loss of H3K27 trimethylation. These tumors characteristically harbor unmethylated MGMT promoters, conferring temozolomide resistance [5]. Additionally, the relatively intact blood–brain barrier limits systemic drug delivery [6,7]. These biological features underscore the need for novel therapeutic paradigms.

### 3.2. Reconsidering Conventional Treatment Paradigms

Unlike supratentorial glioblastoma, extent of resection does not correlate with survival in H3K27M-mutant DMG [8]. Radiotherapy provides modest benefit, typically extending median survival by 3–6 months [9]. These limitations necessitate the exploration of individualized, biology-driven approaches.

### 3.3. Electric Field-Based Therapy

Electric field-based modalities use alternating electric fields to disrupt mitotic spindle formation, interfere with tubulin polymerization, and selectively impair proliferating cells. Tumor Treating Fields (TTFields), an FDA-approved electric field therapy for glioblastoma, delivers intermediate-frequency (200 kHz) alternating electric fields at higher intensity (≥1 V/cm field strength) via transducer arrays applied directly to the scalp, requiring continuous wear (≥18 h daily). Electro-capacitive cancer therapy (ECCT) utilizes a conceptually similar approach but differs in technical implementation: it employs capacitively coupled electrodes rather than insulated transducer arrays, operates at potentially lower field intensities, and delivers electric fields through capacitive coupling rather than direct conductive contact. The precise field strength and distribution achieved with ECCT in clinical use have not been as rigorously characterized as TTFields.

In this case, ECCT was selected based on its accessibility, its lower cost compared to TTFields, and preliminary reports suggesting its potential benefit in pediatric and young adult gliomas. Preliminary data suggest that electric field-based therapies may also modulate the tumor microenvironment and enhance drug delivery, although these findings remain exploratory [10]. Recent case reports have described the potential clinical benefit of integrating electric field-based therapy into multimodal regimens for DMG, meriting further systematic study [11]. However, the evidence base for ECCT remains limited to case reports and small case series, and no randomized controlled trials have been conducted. The relative contribution of ECCT to the overall treatment response in this case cannot be determined.

### 3.4. Dordaviprone and Metabolic Targeting

Dordaviprone (ONC201) is the first systemic agent to receive accelerated FDA approval specifically for H3K27M-mutant DMG. Pooled analyses from five clinical studies including 50 patients reported an overall response rate of approximately 22% [12]. ONC201 is an orally available, blood–brain barrier penetrant small molecule that exerts antitumor effects through enhancement of TRAIL signaling, the activation of the integrated stress response, and antagonism of dopamine D2/3 receptors [13,14]. Recent preclinical work suggests that ONC201 may induce a lineage transition from a proliferative oligodendrocyte-precursor-like state to a more differentiated astrocytic phenotype, promoting terminal differentiation and the loss of tumorigenicity [15].

We combined ONC201 with 2-deoxy-D-glucose, a glycolytic inhibitor that competitively impairs glucose metabolism, resulting in ATP depletion and metabolic stress [16,17]. Beyond its metabolic effects, 2-DDG can inhibit N-linked glycosylation, triggering endoplasmic reticulum stress and the activation of the unfolded protein response [18,19]. In vitro studies indicate that 2-DDG may act synergistically with several anticancer agents, providing a rationale for its inclusion in combination regimens [20].

### 3.5. Precision Medicine and Molecularly Guided Pathway Targeting

The present case illustrates the application of precision oncology principles to H3K27-altered DMG. Comprehensive NGS identified a high-allelic-fraction NF1 mutation, suggesting activation of the RAS/MAPK pathway, while immunohistochemical analysis demonstrated elevated phosphorylated mTOR, indicative of enhanced mTOR signaling within the PI3K/AKT/mTOR axis. These findings provided a mechanistic rationale for incorporating trametinib, a MEK1/2 inhibitor, and everolimus, an mTOR inhibitor, to directly target two convergent signaling hubs [21,22,23].

This dual-pathway inhibition strategy was intended to address key oncogenic drivers and reduce the likelihood of compensatory feedback activation. Both trametinib and everolimus have established clinical use in oncology, relatively favorable CNS penetration, and well-characterized safety profiles [24,25].

### 3.6. Emerging Paradigms: Functional Precision Medicine

Functional precision medicine (FPM) approaches aim to complement genomic profiling with ex vivo drug sensitivity testing (DST) performed on patient-derived tumor cells [26]. In one reported DMG case, DST involving 175 FDA-approved and investigational agents guided the selection of a personalized combination of disulfiram and ONC201 at progression, resulting in radiological response and survival of 15 months after treatment initiation—substantially longer than the approximately 3-month median survival typically observed after progression in DMG [26].

More broadly, the integration of genomic, transcriptomic, proteomic, and functional data within a multi-omics framework may allow for the identification of otherwise unrecognized vulnerabilities and enable a more refined, individualized therapeutic strategy.

### 3.7. Real-World Evidence

Real-world experiences support the feasibility and potential impact of molecular profiling in diffuse intrinsic pontine glioma (DIPG) and DMG. In the INFORM registry, the comprehensive molecular characterization of 21 DIPG cases was achieved with a median turnaround time of 22 days; targetable alterations were identified in 76% of tumors, and five patients received matched targeted therapies [27]. An Italian series of 25 DIPG patients similarly found targetable alterations in 60%, with nine patients receiving molecularly guided treatments. The median overall survival was longer in the targeted therapy group (20.26 months) compared with those who did not receive targeted agents (14.18 months), providing preliminary evidence of a survival benefit associated with precision-medicine-based management [27].

### 3.8. Multimodal Combination Strategies

The favorable clinical course observed in our patient is consistent with emerging reports suggesting that comprehensive multimodal approaches may achieve more durable disease control in DMG. For example, a recent case described a 20-year-old woman with H3K27M-mutant DMG treated with surgery, radiotherapy, chemotherapy, electric field therapy, immunotherapy, and targeted agents, achieving an overall survival of 28 months and a progression-free survival of 21 months, substantially exceeding historical benchmarks [11].

Analyses of ONC201 clinical trial cohorts indicate that the timing of interventions is critical: patients treated after initial radiotherapy but before radiographic recurrence had a median overall survival of 21.7 months, compared with 9.3 months for those who initiated ONC201 after progression [13]. These data support the early integration of molecularly targeted agents rather than reserving them solely for salvage settings. Multiple ongoing trials are exploring rational combination strategies, including a Phase 2 study of ONC201 with panobinostat or paxalisib, and a Phase 3 trial comparing ONC201 to everolimus monotherapy [28]. The results of these trials will be important in clarifying the role of such combinations.

### 3.9. Clinical Response and Implications

In the context of historically poor outcomes for adult H3K27-altered DMG, the substantial radiological response and preserved neurologic function in this case are noteworthy. While causality cannot be definitively established from a single case, the temporal association between treatment initiation and tumor response (combined with the molecular rationale for each component) supports the potential contribution of this individualized approach. The approximate 60% reduction in enhancing tumor volume and sustained clinical stability (Karnofsky Performance Score: 100%) suggest that mechanism-based, genomically guided therapy may favorably alter disease trajectory in selected patients.

This case highlights several translatable principles: the importance of early comprehensive molecular profiling to enable targeted therapy selection; the value of multidisciplinary oversight when integrating approved, off-label, and investigational modalities; and the necessity of proactive toxicity monitoring and shared decision making. While larger prospective studies are required to validate this approach, the favorable outcome demonstrates the feasibility and potential benefit of rationally designed, molecularly informed treatment strategies in this challenging disease.

### 3.10. Limitations and Future Directions

Several limitations warrant emphasis. As a single case, this experience cannot establish causality or generalizability. The observed response may reflect idiosyncratic tumor biology, host factors, or other unmeasured variables. The relative contribution of each component of the regimen cannot be disentangled. While NGS and immunohistochemistry provided a mechanistic framework, we did not perform a functional validation of pathway activation or drug sensitivity.

Longer follow-up is required to assess the durability of response and overall survival. Nonetheless, this case adds to a growing body of evidence suggesting that precision medicine approaches may have a meaningful role in H3K27-altered DMG. The prospective evaluation of individualized, molecularly guided regimens in N-of-1 trials, adaptive platform trials, or basket studies is urgently needed [29]. The incorporation of circulating tumor DNA, advanced imaging biomarkers, and comprehensive multi-omics profiling may further refine patient selection and enable real-time treatment adaptation.

## 4. Conclusions

H3K27-altered diffuse midline glioma remains a highly lethal disease with limited effective treatment options. However, the convergence of advanced molecular profiling, targeted pathway inhibition, metabolic modulation, tissue-agnostic therapies, and electric field-based modalities offers new opportunities to improve disease control and patients’ quality of life.

In this adult patient with H3K27-altered DMG, the implementation of an individualized, genomics-guided, multimodal treatment strategy was associated with substantial radiological regression, sustained neurological stability, and preserved functional status. While isolated observations cannot be generalized, they support further systematic investigation of precision-medicine-based approaches in this tumor type.

Future research should aim to define the therapeutic value of pathway-targeted combinations, identify predictive biomarkers of response and resistance, and determine whether integrated precision oncology can meaningfully alter the natural history of this devastating disease.

## Data Availability

The data presented in this study are available on request from the corresponding author due to the inclusion of personal radiological information in the case report.

## Supplementary Materials

The following supporting information can be downloaded at https://www.mdpi.com/article/10.3390/brainsci16010097/s1. File S1: Complete histopathological examination report with immunohistochemistry and next-generation sequencing analysis; Figure S1: Immunohistochemistry images and H3K27 alteration in the diffuse midline glioma.

## Abbreviations

Alphabetical List of Abbreviations

2-DDG	2-Deoxy-D-glucose
ACMG	American College of Medical Genetics and Genomics
AMP	Association for Molecular Pathology
ATP	Adenosine triphosphate
ATRX	Alpha-thalassemia X-linked intellectual disability syndrome
CBC	Complete blood count
CNS	Central nervous system
CIViC	Clinical Interpretation of Variants in Cancer
CTCAE	Common Terminology Criteria for Adverse Events
DIPG	Diffuse Intrinsic Pontine Glioma
DMG	Diffuse Midline Glioma
DST	Drug Sensitivity Testing
ECC	Electro-Capacitive Cancer Therapy
EMA	Epithelial Membrane Antigen
ETV	Endoscopic Third Ventriculostomy
FDA	Food and Drug Administration (United States)
FPM	Functional Precision Medicine
GFAP	Glial Fibrillary Acidic Protein
Gy	Gray (unit of radiation dose)
H3F3A	H3 Histone Family Member 3A
H3K27M	Histone H3 Lysine 27 to Methionine
H3K27me3	Histone H3 Lysine 27 Trimethylation
IDH	Isocitrate Dehydrogenase
IHC	Immunohistochemistry
KPS	Karnofsky Performance Score
MEK	Mitogen-Activated Protein Kinase Kinase
MGMT	O6-Methylguanine-DNA Methyltransferase
MRI	Magnetic Resonance Imaging
mTOR	Mammalian Target of Rapamycin
NeuN	Neuronal Nuclei
NF1	Neurofibromin 1
NGS	Next-Generation Sequencing
OncoKB	Oncology Knowledge Base
OLIG-2	Oligodendrocyte transcription factor 2
ONC201	Dordaviprone (imipridone-201)
PHH3	Phospho-Histone H3
PI3K/AKT/mTOR	Phosphoinositide 3-Kinase/Protein Kinase B/Mammalian Target of Rapamycin
p-mTOR	Phosphorylated Mammalian Target of Rapamycin
RAS/MAPK	Rat Sarcoma/Mitogen-Activated Protein Kinase
TRAIL	TNF-Related Apoptosis-Inducing Ligand
TTFields	Tumor Treating Fields
VAF	Variant Allele Frequency
WHO	World Health Organization
