# Supplementary material for: Adult-Onset Diffuse Midline Glioma, H3K27-Altered: A Genomics-Guided, Individualized, Multimodal Treatment Approach
Source: Brain Sci. 2026 Jan 16;16(1):97. doi: 10.3390/brainsci16010097 (PMC12838920; doi:10.3390/brainsci16010097)
Supplement: Supplementary file 1 [file brainsci-16-00097-s001.zip › File S1 Complete histopathological examination report with immunohistochemistry and next-generation sequencing analysis.pdf]

**Hasta Adı Soyadı:**	[redacted]
**Hasta Tc No:**	
**Protokol No:**	
**Cinsiyet / D.Tarihi:**	
**Gönderen Bölüm:**	
**Göndere Doktor**	
**Onay Tarihi:**	07.03.2025 14:44:49

ICD-0 Morfoloji 8000/3 - Malign neoplazi

Ön Tanı

J01,R52.9,R68.8,Z01.0,G91,R00,R90,J98.9,E55.9,R68 AKUT SİNÜZİT,AĞRI, TANIMLANMAMIŞ,GENEL SEMPTOM VE BELİRTİLER DİĞER, TANIMLANMIŞ,GÖZ VE GÖRME MUAYENESİ,HİDROSEFALİ,KALP ATIM ANORMALLİKLERİ,MERKEZİ SİNİR SİSTEMİNİN GÖRÜNTÜLEMESİNDE ANORMAL BULGULAR,S

Klinik Bilgi

31Y K HASTA
BİLİNEN MARFAN SEND
KONTRASTLI KRANİAL MR DA T1 İZOİNTENS T2 HİPERİNSTENS HETEROJEN KONTRAST TUTAN TEKTAL PLATE KAYNAKLI HİDROSEFALİYE NEDEN OLAN KİTLE
GLİAL? GERMİNOM? PİNEALOBLASTOM?

Makroskopi

Topluca; 1x0,6x0,2 cm boyutlarında beyaz renkte doku parçasıdır. Doku küçük olduğundan immünhistokimyasal ve moleküler inceleme sonucu doku kaybı yaşanmaması için örnek 2 bloğa bölünmüştür. (A ve B kodlu blok şeklinde)

Mikroskopi

Histomorfolojik Bulgular:

1- Hücresellik artışı: Hafif
2- Atipi ve pleomorfizm: Orta
3- Mitoz: Var (11-12/10 BBA) PHH3 immünhistokimyasal inceleme ile sayıldı
4- Vasküler endotelial proliferasyon (VEP): Yok
5- Nekroz: Yok
6- Mikrokalsifikasyon: Yok
7- Perivasküler lenfositik infiltrasyon: Yok
8- Rosental fibrilleri: Yok
9- Büyüme paterni: İnfiltratif

Ek Parametreler

İmmünohistokimya Paneli: (Bölünmeden önceki parafin bloktan çalışıldı)

- **GFAP (Thermofisher/Astro6) :** Neoplastik hücrelerde immünekspresyon izlendi.
- **EMA (Leica/GP1.4):** Neoplastik hücrelerde immünekspresyon izlenmemiştir.
- **OLIG-2 (CellMarque/211F1.1):** Neoplastik hücrelerde immünekspresyon izlendi.
- **Ki67 (Zeta-MIB1):** Proliferatif indeks yaklaşık %15'dir.
- **Kromogranin (CellMarque/SP12):** Neoplastik hücrelerde immünekspresyon izlenmemiştir.
- **Sinaptofizin (Leica/27G12):** Neoplastik hücrelerde immünekspresyon izlenmemiştir.
- **CD34 (scytek QBEnd/10):** Neoplastik hücrelerde immünekspresyon izlenmemiştir.
- **NeuN (Miliqore/A60):** Neoplastik hücrelerde immünekspresyon izlenmemiştir, Nöronlarda immünekspresyon izlenmiştir.
- **NFP (CellMarque/Ep79):** İnfiltratif özellikte tümör izlendi.

"Bu raporda yer alan bilgiler MÜFT Patoloji AD / Kliniği onayı olmadan araştırma ve yayın amaçlı kullanılamaz"

**Hasta Adı Soyadı:**	[redacted]
**Hasta Tc No:**	
**Protokol No:**	
**Cinsiyet / D.Tarihi:**	
**Gönderen Bölüm:**	
**Göndere Doktor**	

ICD-0 Morfoloji 8000/3 - Malign neoplazi

İmmünohistokimya Paneli: **(A kodlu parafin bloktan çalışıldı)**

- **PHH3 (CellMarque/Poliklonal):** Mitoz 11-12/10BBA izlendi.
- **IDH(DİONOVA/H09):** Neoplastik hücrelerde immünekspresyon izlenmemiştir.(Eksternal kontrol çalışılmıştır).
- **H3K27M (millipore polyclonel):** Dağınık az sayıda hücrede immünekspresyon izlendi.
- **H3K27M-me3 (Biosciences/RM175):** Fokal alanda az sayıda hücrede şüpheli immünekspresyon kaybı izlenmiştir.

Tanı

Diffüz infiltratif glial tümör, lütfen notu okuyunuz; "tektal plate kaynaklı kitle" kodlu biyopsi.

Not-Açıklama

1-) Mevcut biyopsi örneğinde az sayıda küçük fragmanlar halinde gri ve beyaz cevhere ait dokular görülmektedir. Mevcut fragmanların birkaçında selülerite görece artmış olup yer yer (bir kısmı iğsi hücreli görünümde) pleomorfik atipik hücreler görülmektedir. Uygulanan immünhistokimyasal incelemelerde bu hücrelerin GFAP ve OLİG ile pozitif, sinaptofizin ve kromogranin ile negatif olduğu görülmüştür. Mevcut örneklerin Hematoksilen&eozin kesitlerinde nekroz, vasküler endotelyal proliferasyon ve mitoz saptanmamış olup uygulanan PHH3 immünhistokimyasında 10 BBA'da 11-12 adet mitotik figür saptanmıştır. Ki-67 proliferasyon indeksi de yaklaşık %15 olarak değerlendirilen olguda başta H3K27 alterasyonu gösteren diffüz orta hat gliomu ve yüksek dereceli astrositomlar ayırıcı tanıya alınmıştır. İmmünhistokimyasal olarak IDH negatif saptanmıştır. H3K27M ile dağınık az sayıda hücrede immünekspresyon görülmüş olup H3K27me3 ile zayıf boyanma/kayıp? tarzında şüpheli bulgular saptanmıştır.
2-) Olguda IDH1 immünhistokimyasal olarak negatiftir. DSÖ 2021 Santral sinir sistemi tümör sınıflamasına göre IDH mutasyonu immünhistokimyasal olarak olguların %85'inde saptanabilmektedir. 55 yaşının altındaki olgularda, Dünya Sağlık Örgütü(DSÖ) IDH1 ve IDH2 mutasyonlarının moleküler olarak olarak (PCR/dizi analizi) ile incelenmesini önermektedir. Ayrıca H3K27m ve H3K27Me3 immünhistokimyasında da bulgular şüpheli olarak değerlendirildiğinden kesin tanı ve derecelendirmenin NGS sonuçlarıyla birlikte verilmesi uygundur. NGS sonucu ek raporla bildirilecektir.

Ek Rapor

27/03/2025 TARİHLİ EK RAPOR;

Olguya ait LİOS sistemi üzerindeki **M25-YPATO-283** kod ile kayıtlı olan Yeni Nesil Dizileme (YND / NGS) raporuna göre ;

Yöntem: Kapsamlı Merkezi Sinir Sistemi Paneli

SONUÇ / YORUM: Hastanın DNA materyalinde, Tier-I sınıfında, **ATRX** geninde c.1443_1452del p.Glu482GlnfsTer29 frameshift deg?is?imi alel fraksiyonu %34.4 olarak go?zlenmektedir. ATRX geninde go?zlenen kısa protein olus?umuna neden olan mutasyonlar CNS'de genellikle IDH mutasyonları ile go?ru?lu?r ve tu?mo?rde saptanan dig?er deg?is?imlerle birlikte diagnostik o?nemi bulunmaktadır [NCCN-CNS cancers, March 2025; PMID: 23530248]. Bu deg?is?imin dig?er deg?is?imler ve hastanın dig?er tetkik sonuçları ile deg?erlendirilmesi o?nerilir.

Hastanın DNA materyalinde, Tier-I sınıfında, **H3F3A** geninde c.83A>T p.(Lys28Met) (K27M) missense deg?is?imi %27.2 oranında go?zlenmektedir. Literatu?rde histone H3K27M olarak geçen bu deg?is?im (doi: 10.1002/path.5666) beyin tu?mo?rlerinde en sık go?ru?len deg?is?im olup H3.3 histone trimetilasyonunu engelledig?i bilinmektedir. NCCN'e go?re bu deg?is?im infiltrative glioma diagnozu için bir kanıt olup bu deg?is?imi tas?ıyan hastalarda daha ko?tu? prognoz go?zlenmektedir [NCCN-CNS cancers,

Hasta Adı Soyadı:	[redacted]
Hasta Tc No:	
Protokol No:	
Cinsiyet / D.Tarihi:	
Gönderen Bölüm:	
Göndere Doktor	

ICD-0 Morfoloji 8000/3 - Malign neoplazi

March 2025]. **WHO sınıflandırmasına go?re H3K27 deg?is?imi go?ru?len tu?mo?rler "diffu?z midline gliomu, WHO grade 4" olarak sınıflandırılmaktadır [WHO-Classification of tumors of the CNS, 2021, PMID: 27157931].**

Hastanın DNA materyalinde, Tier-I sınıfında, **NF1** geninde c.586+1G>C splice site deg?is?imi alel fraksiyonu %64.1 olarak gözlenmektedir. NF1 deg?is?imleri astrositom ve gliono?ral tu?mo?rlerde go?ru?lmekte [WHO-CNS, 2021]. NF1-mutant gliomalarda MEK inhibito?ru? selumetinib'e duyarlılık go?ru?lebilecg?ei bildirilmektedir [NCCN-CNS cancer, January 2025]. Literatürde, H3K27 deg?is?imi saptanan diffu?z midline gliom hastalarında NF1 ve ATRX deg?is?imlerinin de birlikte go?zlemlendig?i belirtilmis?tir (PMID: 37524847, 29063183). Hastanın detaylı klinik bilgisi ile deg?erlendirilmesi o?nerilir.

Archer MSI algoritmasına go?re hastanın MS durumu "**MS-Stabil**" olarak tespit edilmis?tir.

NOT: Hastanın dokusu küçük olduğundan ve çalışma öncesi kalite metriklerini geçemediğinden RNA izolasyonu yapılamamıştır ve bu çalışmada RNA verisi değerlendirilememiştir.

Bu sonuçlara göre olgu "H3K27 alterasyonu gösteren diffüz orta hat gliomu, DSÖ grade 4" olarak değerlendirilmiştir.

Ayrıntılı sonuç için lütfen LIOS sisteminde kayıtlı rapora bakınız.

Asistan Dr. SEHER EDA HOROZ
Tıbbi Patoloji

Uzm.Dr. BUKET GEDİK
Tıbbi Patoloji Uzmanı

"Bu raporda yer alan bilgiler MÜFT Patoloji AD / Kliniği onayı olmadan araştırma ve yayın amaçlı kullanılamaz"
