# Supplementary figures and images for: Adult-Onset Diffuse Midline Glioma, H3K27-Altered: A Genomics-Guided, Individualized, Multimodal Treatment Approach
Source: Brain Sci. 2026 Jan 16;16(1):97. doi: 10.3390/brainsci16010097 (PMC12838920; doi:10.3390/brainsci16010097)

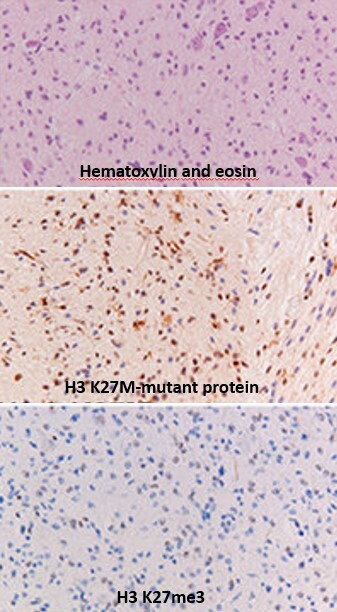

Supplement: Supplementary file 1 [file brainsci-16-00097-s001.zip › Figure S1 Immunohistochemistry images, H3K27 alteration in the diffuse midline glioma..jpg]
